# An annotated chromosome-level genome for the red-fronted brown lemur (*Eulemur rufifrons*) sheds light on brown lemur evolution

**DOI:** 10.1093/g3journal/jkaf213

**Published:** 2025-09-11

**Authors:** Kathryn M Everson, Andrew N Black, Mariah E Donohue, David W Weisrock

**Affiliations:** Department of Integrative Biology, Oregon State University, Corvallis, OR 97330, United States; Center for Quantitative Life Sciences, Oregon State University, Corvallis, OR 97330, United States; Department of Biology, University of Kentucky, Lexington, KY 40506, United States; Department of Biological Sciences, Binghamton University, State University of New York, Binghamton, NY 13902, United States; Department of Biology, University of Kentucky, Lexington, KY 40506, United States

**Keywords:** conservation, genome assembly, Hi-C, lemur, Madagascar, Pacific biosciences

## Abstract

The red-fronted brown lemur (*Eulemur rufifrons*) is an important species to the function of Madagascar's ecosystems, contributing to critical ecological processes such as seed dispersal. Given its ecological, as well as cultural, importance, genomic resources for *E. rufifrons* are valuable for understanding evolutionary history and informing conservation strategies. In this study, we present an annotated chromosome-level genome assembly for *E. rufifrons*, generated using PacBio HiFi long reads and Hi-C proximity ligation data, and demonstrate its utility for understanding patterns of divergence among members of the genus *Eulemur*. The chromosome-level genome size is 2.41 Gb, and it exhibits high-quality metrics including a scaffold N50 of 100.8 Mb and a BUSCO completeness score of 95.3%. Comparative analyses reveal remarkable synteny between our *E. rufifrons* assembly and the previously published *Eulemur mongoz* genome, despite an estimated divergence of ∼4 million years. Phylogenetic and network analyses identify pervasive signals of gene flow across *Eulemur*, with our focal individual showing unexpected genomic affinities to *Eulemur rufus*, highlighting the need for methods that account for reticulation in phylogenomic studies. Overall, this genome for *E. rufifrons* provides a valuable resource for exploring lemur evolutionary history, genomic divergence, and patterns of hybridization. Future research should leverage chromosome-level assemblies to investigate gene flow, adaptive introgression, and regions under selection, advancing our understanding of lemur diversity and informing conservation strategies for these imperiled primates.

## Introduction

Madagascar hosts a unique assemblage of endemic mammals, including over 113 species of lemurs, which enrich the island with key ecosystem services, including seed dispersal and pollination (Razafindratsima 2015). They also hold important cultural value to local Malagasy people (Loudon et al. 2006) and fuel Madagascar's ecotourism industry. Nonetheless, lemurs face imminent threats from deforestation, climate change, and hunting, with at least 90% of species considered threatened with extinction (Schwitzer et al. 2014; IUCN 2025).

High-quality genomic resources can both enhance lemur conservation and help elucidate evolutionary dynamics in adaptive radiations. Here, we focus on Madagascar's true lemurs (genus *Eulemur,* 12 species), which experienced recent and rapid diversification within the last 5 million years, and exhibit considerable ecological and behavioral diversity within and across species (Mittermeier et al. 2010). *Eulemur* species inhabit extreme environments across Madagascar, from semi-arid spiny forests to lush rainforests. This ecological variation is punctuated with incredible variation in social systems, activity patterns, and diet both within and across species. Genetic data suggest a history of reproductively weak species boundaries in *Eulemur* (Markolf et al. 2013; Everson et al. 2023), with hybridization potentially driving high rates of speciation (Everson et al. 2025).

**Fig. 1.**
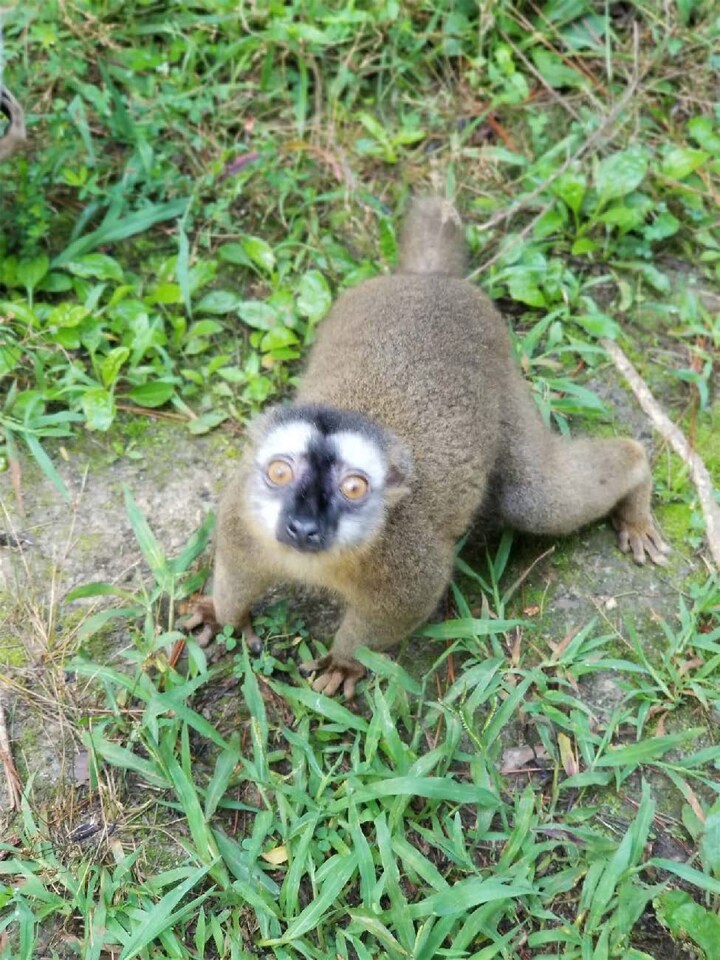
Photo of the source individual for our *E. rufifrons* genome assembly, a captive female from the Duke Lemur Center named Redbay. Photo credit: Duke Lemur Center.

The red-fronted brown lemur (*Eulemur rufifrons*; Fig. 1) is a component of the brown lemur complex (Wyner et al. 1999; Groves 2001), a radiation of 7 lineages that geographically span much of Madagascar and diverged within the last 2 million years (Markolf and Kappeler 2013; Everson et al. 2025). Populations of *E. rufifrons* are divided between eastern rainforests and western dry forests. Western populations tend to have lighter coloration, higher population density, and more generalized diets (Ganzhorn et al. 1999; Mittermeier et al. 2010; Murillo et al. 2022). They also have smaller home ranges and less habitat connectivity (Mittermeier et al. 2010). Eastern populations tend to rely more heavily on fruit, but fall back on leaves during times of resource scarcity (Overdorff 1993, 1996a, 1996b; Sato et al. 2015). Growing evidence suggests that these populations are quite distinct genetically as well (Donohue 2024). *Eulemur rufifrons* has been the topic of several ecological and hybridization-related studies. Of particular interest is a large, stable hybrid zone between *Eulemur cinereiceps* and eastern *E. rufifrons*, a potentially genetically stable population (Wyner et al. 2002) hypothesized to be a new hybrid lineage (Delmore et al. 2013). Recent population genomic data points to hybrid backcrossing with eastern *E. rufifrons* (Donohue 2024), suggesting a more complex scenario of hybridization.

Herein, we present a nearly complete annotated chromosome-scale genome assembly for a female brown lemur from the Duke Lemur Center, named Redbay. Redbay was born in captivity on March 24, 1995, but further information about her pedigree has been lost, so her precise origins and family history are unknown. However, based on morphology and coloration, she was likely descended from a western *E. rufifrons* population (Fig. 1). Her genome was sequenced using PacBio long reads and Hi-C proximity ligation. We evaluate synteny between this new genome and a previously published chromosome-scale genome of the mongoose lemur (*Eulemur mongoz*). We also use previously published scaffold-level genomes to estimate phylogenetic relationships, genomic divergence, and patterns of gene flow across the genus *Eulemur*.

## Materials and methods

### Sample collection and sequencing

The 27-yr-old captive female *E. rufifrons* individual named Redbay was euthanized by a veterinarian in January 2023 due to health issues in old age. During her necropsy, 5 aliquots (0.25 g) each of heart, kidney, and blood were collected for genomic DNA. Aliquots were flash frozen with liquid nitrogen, temporarily stored in a −80 °C freezer at the DLC, and then shipped on dry ice to Oregon State University.

Genomic DNA (gDNA) extraction was performed in the Center for Quantitative Life Sciences at Oregon State University using a Nanobind High Molecular Weight DNA Extraction kit (Circulomics, Inc.). Extraction of kidney tissue yielded >15 μg of double-stranded DNA (assessed using a Qubit Fluorometer; Invitrogen, Inc.) and a modal fragment size > 60 kb (assessed using a TapeStation; Agilent, Inc.). Library preparation and sequencing were conducted at the University of Oregon's Genomics and Cell Characterization Core Facility. Briefly, gDNA was sheared to an average fragment length of 13 kb using a Megaruptor 3 (Diagenode), then sheared fragments were converted to libraries following standard protocols of the SMRTBell Express Template prep kit (3.0). Libraries were sequenced across 6 Sequel II SMRTcells. A Phase Genomics kit was used for the Hi-C library preparation, which was sequenced on an Illumina NovaSeq 6000 platform using 2 × 150 bp sequencing chemistry. Prior to Hi-C sequencing, library fragment sizes were assessed using an Advanced Analytical Fragment Analyzer (Agilent Technologies, Inc.) and quantified via real-time PCR on a QuantStudio instrument (Applied Biosystems, Inc.).

### De novo genome assembly and annotation

PacBio Circular Consensus Sequences (CCS) from both SMRTcells were generated using SMRTLink (v.11.0) with the following parameters: ccs --min-passes 3 --min-rq 0.99. Adapters were then removed using HifiAdapterFilt (Sim et al. 2022). To inform genome assembly and provide context for diversity metrics, “k-mer” counting was conducted with jellyfish (v.2.30; Guillaume and Kingsford 2011) using canonical (-C) 60-mers among Q20 reads. A histogram of “k-mer” counts was then used to estimate genome assembly size and heterozygosity with GenomeScope2 (Ranallo-Benavidez et al. 2020).

Following k-mer assessment, hifiasm (Cheng et al. 2021) was used to produce haplotype-resolved assemblies by incorporating Hi-C reads and Q20 CCS from the 6 SMRTcells. The primary assembly was converted from GFA to FASTA file format and used as a target reference for the alignment of Illumina Hi-C reads. Mapping of Hi-C reads to the indexed primary assembly was done using the Burrow-Wheeler Aligner's mem algorithm (Li and Durbin 2009), allowing a large distance between paired reads (-5SP). Duplicates were removed using samblaster (Faust and Hall 2014). The program samtools (Li et al. 2009) was used to remove supplemental and unmapped reads using bitflags (2316) and alignments with mapping scores below 20. Scaffolding of the assembled contigs was then conducted using yahs (Zhou et al. 2023), specifying the alignment file and primary assembly. Minimap2 (Li 2018) was used to perform genome-genome alignment (-ax asm5) with the chromosome-level *E. mongoz* assembly (GCA_028534055.1; DNA Zoo 2023) to determine synteny and label scaffolds according to chromosome; we note that the *E. mongoz* genome assembly does not include sex chromosomes, thus our chromosomes are similarly labeled (1-30). The genome-genome alignment was visualized using D-Genies (Cabanettes and Klopp 2018). Identified sequences were then reoriented according to the *E. mongoz* chromosome orientation (by taking the reverse complement) and final Hi-C contact maps were visualized using juicebox (v1.9.8; Durand et al. 2016). The *E. rufifrons* mitochondrial genome was assembled using *Lemur catta* as a reference (NC_059325.1; Vertebrate Genomes Project 2021) in the program mitohifi (Uliano-Silva et al. 2023). Final genome assembly metrics were generated using quast (Gurevich et al. 2013). Gene annotation was completed using the NCBI Eukaryotic annotation pipeline (Thibaud-Nissen et al. 2013). Briefly, this pipeline includes the following steps: (i) masking of repetitive elements using WindowMasker (Morgulis et al. 2006), (ii) alignment of known RefSeq transcripts from closely related species using Splign (Kapustin et al. 2008), (iii) gene prediction using Gnomon (Souvorov et al. 2010), and (iv) assignment of Gene Ontology (GO) terms for all annotated proteins using InterProScan (Jones et al. 2014). Assembly completeness was evaluated with Benchmarking Universal Single-Copy Orthologs (BUSCO) using 13,780 orthologs in the Primate database (v. 5.4.1; Manni et al. 2021). To provide context, all other *Eulemur* assemblies available on NCBI as of April 2025 (n = 15; University of Chicago 2015a, 2015b; Broad Institute 2019; DNA Zoo 2023; Institut de Biologia Evolutiva 2023a, 2023b, 2023c, 2023d, 2023e, 2023f, 2023g, 2023h, 2023i, 2023j; Rutgers, The State University of New Jersey 2025) and the *L. catta* assembly (Vertebrate Genomes Project 2021) were downloaded and analyzed using the same BUSCO parameters.

### Repetitive element analyses

We analyzed the repeat content in our *E. rufifrons* genome using the program RepeatModeler (v.1.0.11; Flynn et al. 2020) with 5 rounds of analysis under default settings. The resulting repeat library was then used as input for RepeatMasker (v.1.2.09; http://repeatmasker.org) using the RMBlast model for alignment. We also used the program tidk (Brown et al. 2025) to identify telomeric repeats within all assembled chromosomes and unplaced scaffolds. Specifically, we searched for the mammalian (TTAGGG)_n_ telomeric repeat (Weber et al. 1991) in 10 kb windows across the assembly using the “tidk search” command.

### Synteny analysis

Synteny was visualized between our *E. rufifrons* assembly and the previously published chromosome-scale assembly of *E. mongoz* (GCA_028534055.1) following the protocol outlined in Manni et al. (2021). Briefly, the input for this protocol is the “full_table.tsv” file generated by BUSCO in the previous step, which contains information on the genomic positions of each single-copy ortholog. The plots were generated using the snakemake and R scripts distributed with Manni et al. (2021) in the support_protocol2/plot_syntenies directory.

### Phylogenetic tree and network analyses

To estimate a phylogeny for *Eulemur*, nucleotide sequences from orthologous, single-copy genes were first extracted from our BUSCO results directories (n = 17) to produce 1 phylip-formatted alignment file for each ortholog using MAFFT v7.520 (Katoh and Standley 2013) under default settings. Individual gene trees were then estimated for each alignment via maximum-likelihood inference in RaxML-ng v1.2.0 (Kozlov et al. 2019) using a generalized time-reversible substitution model with rate heterogeneity (-m GTRCAT). Finally, this set of gene trees was used to estimate a phylogeny via ASTRAL-IV, a quartet-based program that is robust to gene tree discordance caused by incomplete lineage sorting (Mirarab et al. 2014; Zhang and Mirarab 2022). ASTRAL-IV measures branch support as local posterior probabilities (Sayyari and Mirarab 2016), and we also calculated gene and site concordance factors (CFs) on each node using the fixed ASTRAL-IV topology in IQTree2 (Minh et al. 2020) via the commands --gcf and --scf, respectively. We visualized the final phylogeny using FigTree (http://tree.bio.ed.ac.uk/software/figtree/).

The same gene tree set was also used as input for phylogenetic network inference in SNaQ, implemented in the Julia package PhyloNetworks (Solís-Lemus et al. 2017). This method uses the multispecies coalescent to estimate a species tree under models with a user-defined number of reticulate branches (*H*). Networks were explored with up to 5 reticulations (*H* = 0 to 5), reporting the network with the lowest pseudolikelihood score, and were visualized using IcyTree (Vaughan 2017). We note that in this analysis, our *E. rufifrons* genome was treated as a distinct “species” compared to the other previously published *E. rufifrons* genome (GCA_043251655), as these 2 individuals were not recovered as a monophyletic group in our ASTRAL-IV analysis (see Results).

## Results

### De novo genome assembly and annotation

**Fig. 2.**
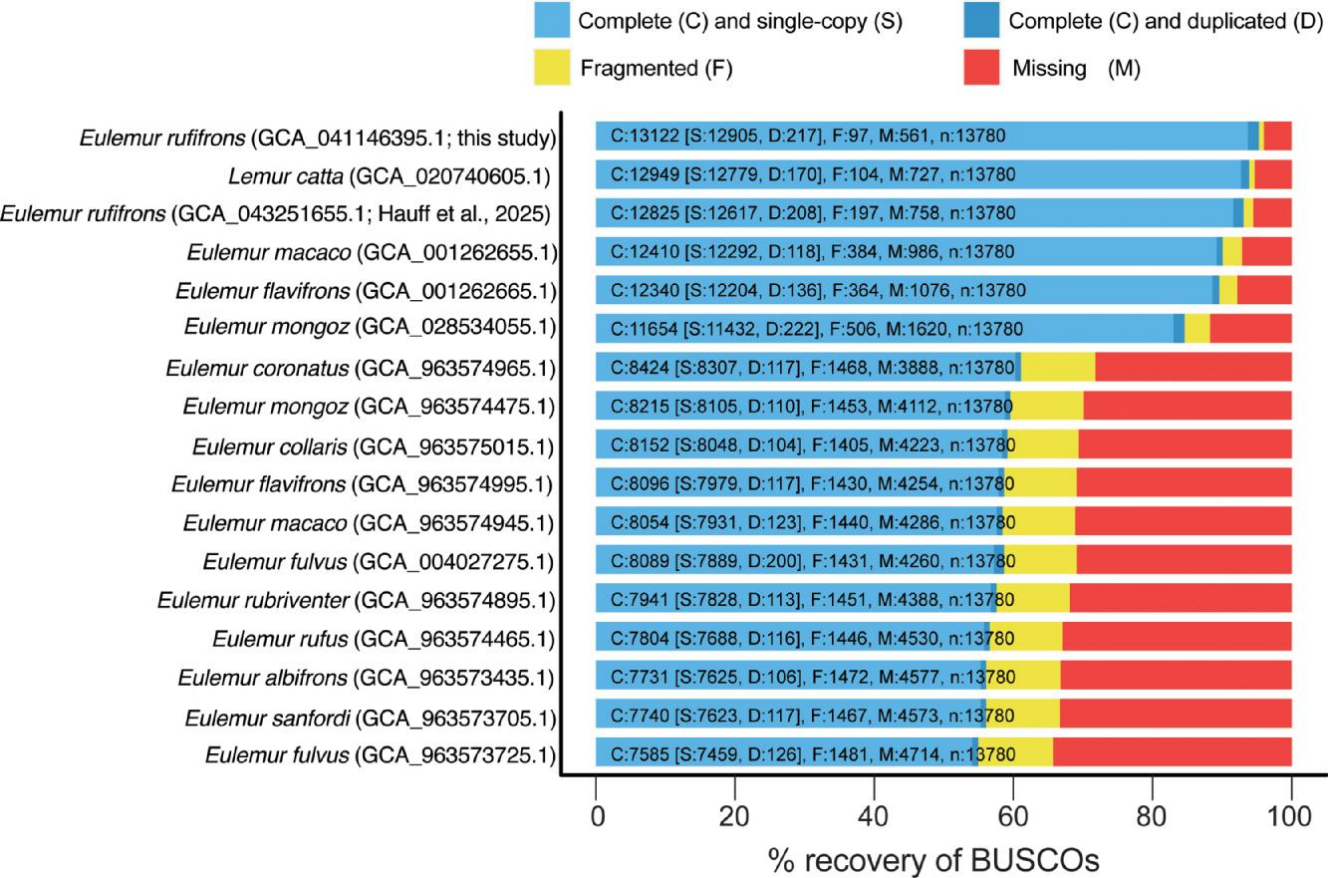
Benchmarking Universal Single-Copy Orthologs (BUSCO) metrics for the *E. rufifrons* assembly, compared to other related published reference assemblies. GenBank numbers are provided for each assembly.

Six PacBio SMRTcells yielded 89.7 Gb of nucleotide data among 9.5 M reads with a mean read length of 16.5 kb. Analysis of 60-mers in the Q20 CCS reads predicted a 2.01 Gb haploid genome length, with an estimated heterozygosity of 0.56% (i.e. ∼0.56% of the predicted ∼2.01 Gb was heterozygous). Overall, very few adapters were identified and removed among the 6 CCS subreads (i.e. 22 adapter contaminated reads). Following adapter removal, the initial hifiasm primary assembly resulted in in 564 contigs with a N50 of 91 Mb, a mean depth of coverage of 15×, and a genome size of 2.41 Gb. Overall alignment rates of the Hi-C reads was 99%. Following Hi-C scaffolding with yahs, and removal of 2 partial mitochondrial sequences, there were 647 scaffolds with a N50 of 100.8 Gb and a total size of 2.41 Gb (Table 1, Supplementary Fig. 1). Comparative analyses with the *E. mongoz* chromosome assembly identified all 30 autosomes in the *E. rufifrons* assembly, with a close agreement in chromosome similarity (Supplementary Fig. 2) and length (Supplementary Fig. 3). BUSCO analysis of nucleotide sequence data identified 95.3% (single copy: 93.7%, duplicated: 1.6%, fragmented: 0.7%, missing: 4.0%) of the 13,780 orthologs in the genome assembly (Fig. 2). The reconstructed mitochondrial genome was 17,079 nucleotides in length with a GC content of 39%, similar to the ring-tailed lemur mitogenome (length = 17,086, GC = 38%). The NCBI Eukaryotic Genome Annotation Pipeline masked 35.41% of the genome as repetitive elements using WindowMasker (but see our own analysis using RepeatMasker below). From the remaining unmasked regions, 19,514 total protein coding genes were identified with 41,715 mRNA transcripts (Table 1). Of these, 97.1% of the 13,780 orthologs were identified as complete (96.0% single copy, 1.0% duplicated), 0.9% were fragmented, and 2.0% were missing.

**Table 1.** Curated genome assembly and annotation metrics of the *E. rufifrons* assembly.

Assembly name	OSU_ERuf_1
Accession	GCA_041146395.1
Pseudochromosomes	30
Scaffolds	647
N50 (Mb)	100.8
Haploid length (Gb)	2.41
Repetitive elements (WindowMasker)	35%
GC content	41.5%
Mitochondrion length (bp)	17,086
Mitochondrion GC content	38%
Genome coverage	15×
Protein coding genes	19,514
Coding mRNA	41,715
Complete, single copy	96%
Complete, duplicated	1%
Fragmented	1%
Missing	2%

### Repetitive element analyses

RepeatModeler and RepeatMasker identified several distinct families of repetitive elements which made up 33.2% of the genome (Table 2), which is similar to the repeat content percentage identified by WindowMasker in the NCBI Eukaryotic Genome Annotation Pipeline above (35.41%). This overall repeat content is also on par with other lemurs, as reported in the NCBI Annotation Reports of the mouse lemur *Microcebus murinus* (33.75%; GCF_040939455.1) and the ring-tailed lemur *L. catta* (32.32%; GCF_020740605.2). In our genome, long and short interspersed nuclear elements (LINEs and SINEs) were the most common types of repetitive elements, representing 9.81% and 4.76% of the genome, respectively. Our tidk analysis also identified 62.68 Mb of telomeric repeats, of which 51.25 Mb were located within the unplaced scaffolds. Despite the difficulty of assembling telomeric content, we found that 16 of our pseudochromosomes were capped on both ends by telomeric repeats and an additional 11 were capped on 1 end, suggesting that these represent complete or nearly complete chromosomes (Supplementary Fig. 4).

**Table 2.** Repetitive elements in the *E. rufifrons* genome.

	Number of elements	Length occupied (bp)	Percentage of sequence
Retroelements	1,112,125	368,843,357	15.27
SINEs	488,684	114,873,920	4.76
Penelope	0	0	0
LINEs	597,686	237,071,369	9.81
CRE/SLACS	0	0	0
L2/CR1/Rex	71,326	14,809,267	0.61
R1/LOA/Jockey	0	0	0
R2/R4/NeSL	0	0	0
RTE/Bov-B	30,886	3,383,393	0.14
L1/CIN4	495,474	218,878,709	9.06
LTR elements	25,755	16,898,068	0.7
BEL/Pao	0	0	0
Ty1/Copia	0	0	0
Gypsy/DIRS1	825	75,121	0
Retroviral	24,930	16,822,947	0.7
DNA transposons	70,452	30,394,421	1.26
hobo-Activator	24,900	10,254,411	0.42
Tc1-IS630-Pogo	44,348	19,658,954	0.81
En-Spm	0	0	0
MULE-MuDR	729	314,751	0.01
PiggyBac	277	148,508	0.01
Tourist/Harbinger	0	0	0
Other (Mirage, P-element, Transib)	0	0	0
Rolling-circles	0	0	0
Unclassified	1,152,435	291,582,314	12.07
Total interspersed repeats		690,820,092	28.6
Small RNA	11,556	7,325,189	0.3
Satellites	0	0	0
Simple repeats	566,802	87,416,068	3.62
Low complexity	101,386	16,267,645	0.67

### Synteny analysis

**Fig. 3.**
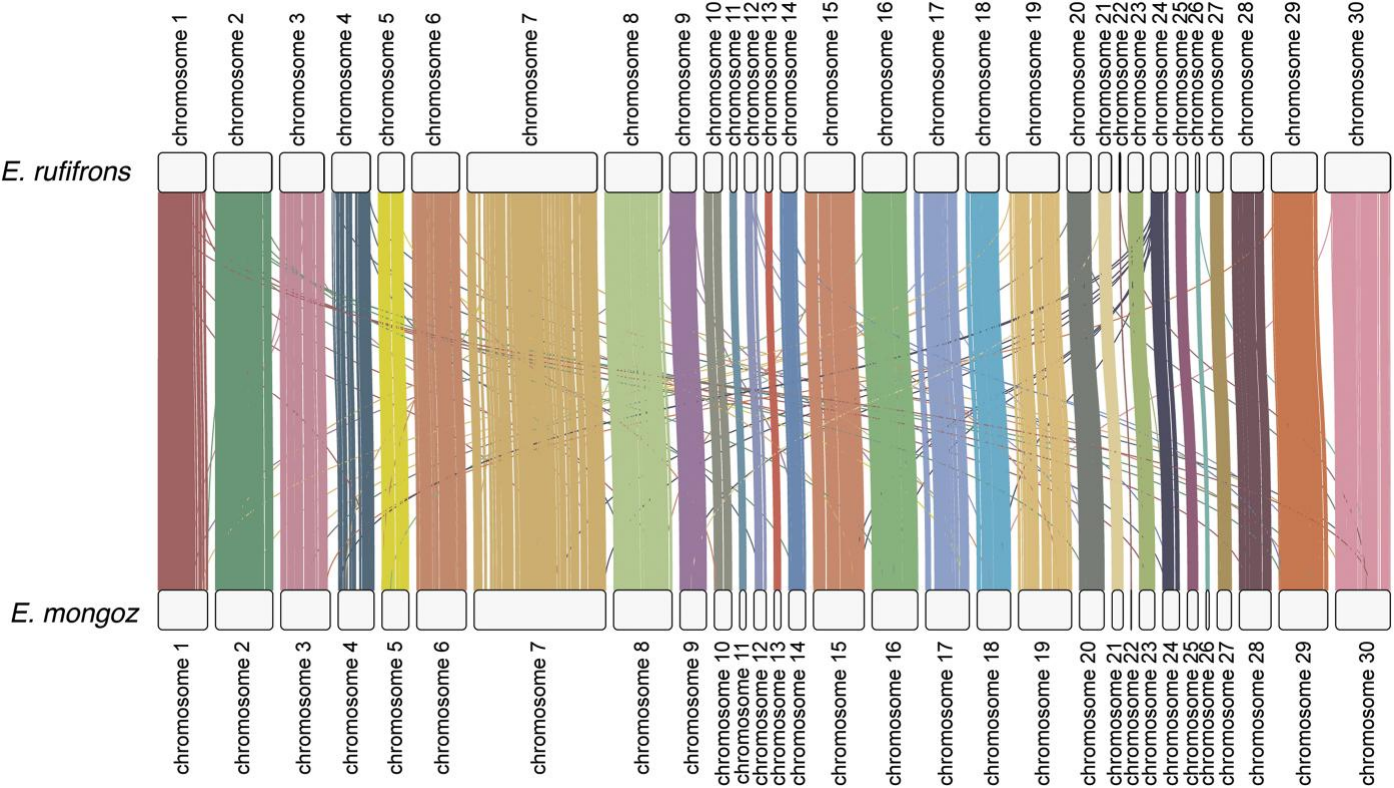
Synteny plot comparing our chromosome-scale *E. rufifrons* genome (GCA_963574465.1) and the *E. mongoz* chromosome-scale genome (GCA_028534055.1). Vertical lines connect the chromosomal positions of BUSCO orthologs in each genome.

Our synteny analysis revealed that the number, size, and order of genes along chromosomes are well conserved between *E. rufifrons* and *E. mongoz* overall (Fig. 3). Of all single-copy BUSCO orthologs we identified, 99.4% are located on the same chromosome in both species.

### Phylogenetic tree and network analyses

**Fig. 4.**
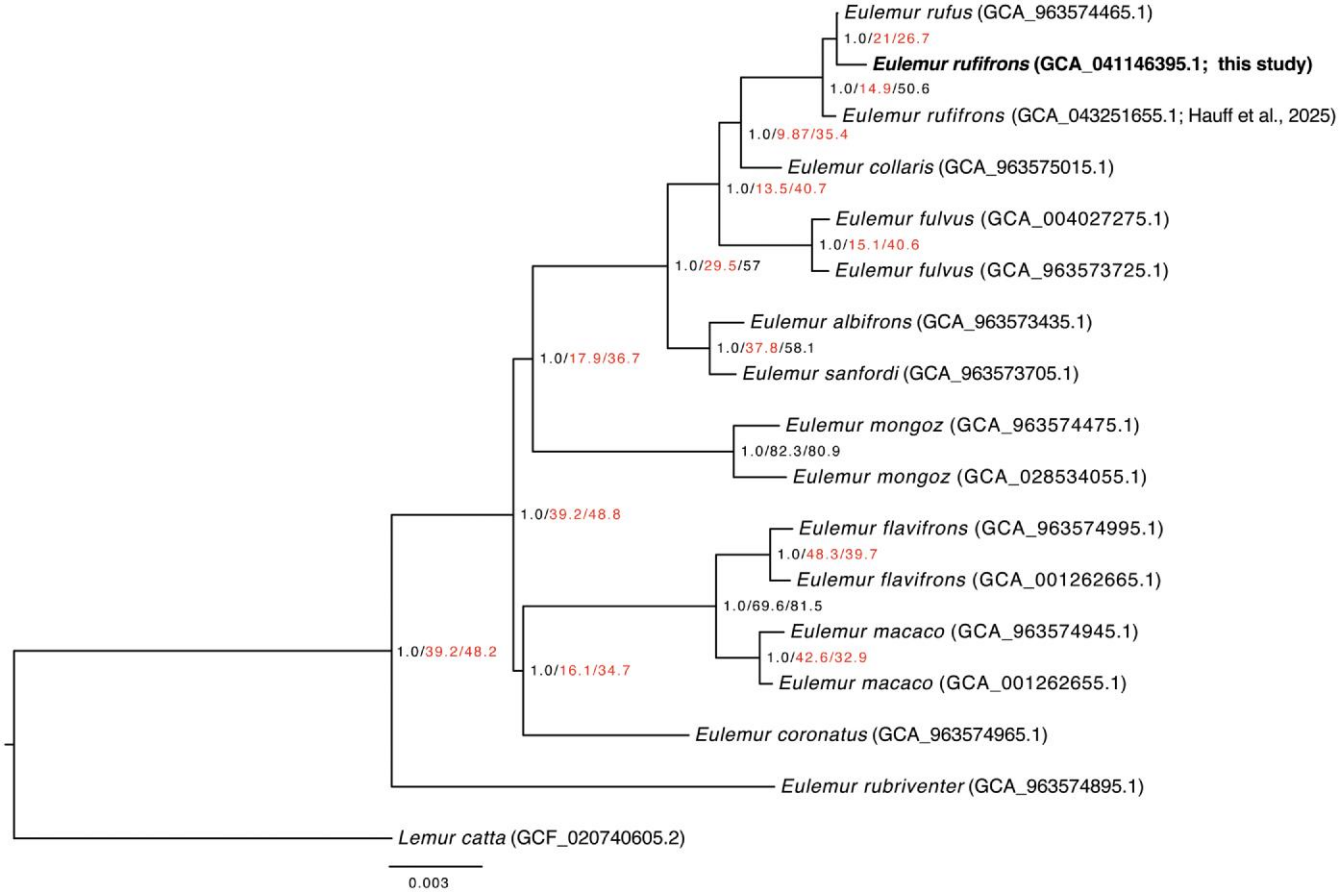
Phylogeny based on BUSCO single-copy genes estimated using ASTRAL-IV. Nodes are labeled with posterior probabilities/gene concordance factors/site concordance factors (CFs), with CFs calculated using IQTree2. Branch metrics with proportions/percentages < 50 are highlighted. Branch lengths are in coalescent units.

Using ASTRAL-IV, we recovered a phylogeny with 1.0 local posterior probabilities on all branches (Fig. 4); however, gene and site concordance factors (gCFs and sCFs) were very low throughout the tree (gCF < 50 on 86.7% of nodes, and sCFs < 50 on 66.7% of nodes), suggesting substantial discordant phylogenetic signal in our data. Values were particularly low for the placement of *Eulemur coronatus* (gCF = 16.1, sCF = 34.7) and *E. mongoz* (gCF = 17.9, sCF = 36.7), and for all relationships within the *Eulemur fulvus* species complex (gCF range = 9.87 to 21.0, sCF range = 26.7 to 50.6). Intriguingly, our focal species, *E. rufifrons*, was not recovered as monophyletic in this analysis; our genome was recovered as sister to *Eulemur rufus* (GCA_963574465), with this clade then being sister to the other previously published *E. rufifrons* (GCA_043251655).

**Fig. 5.**
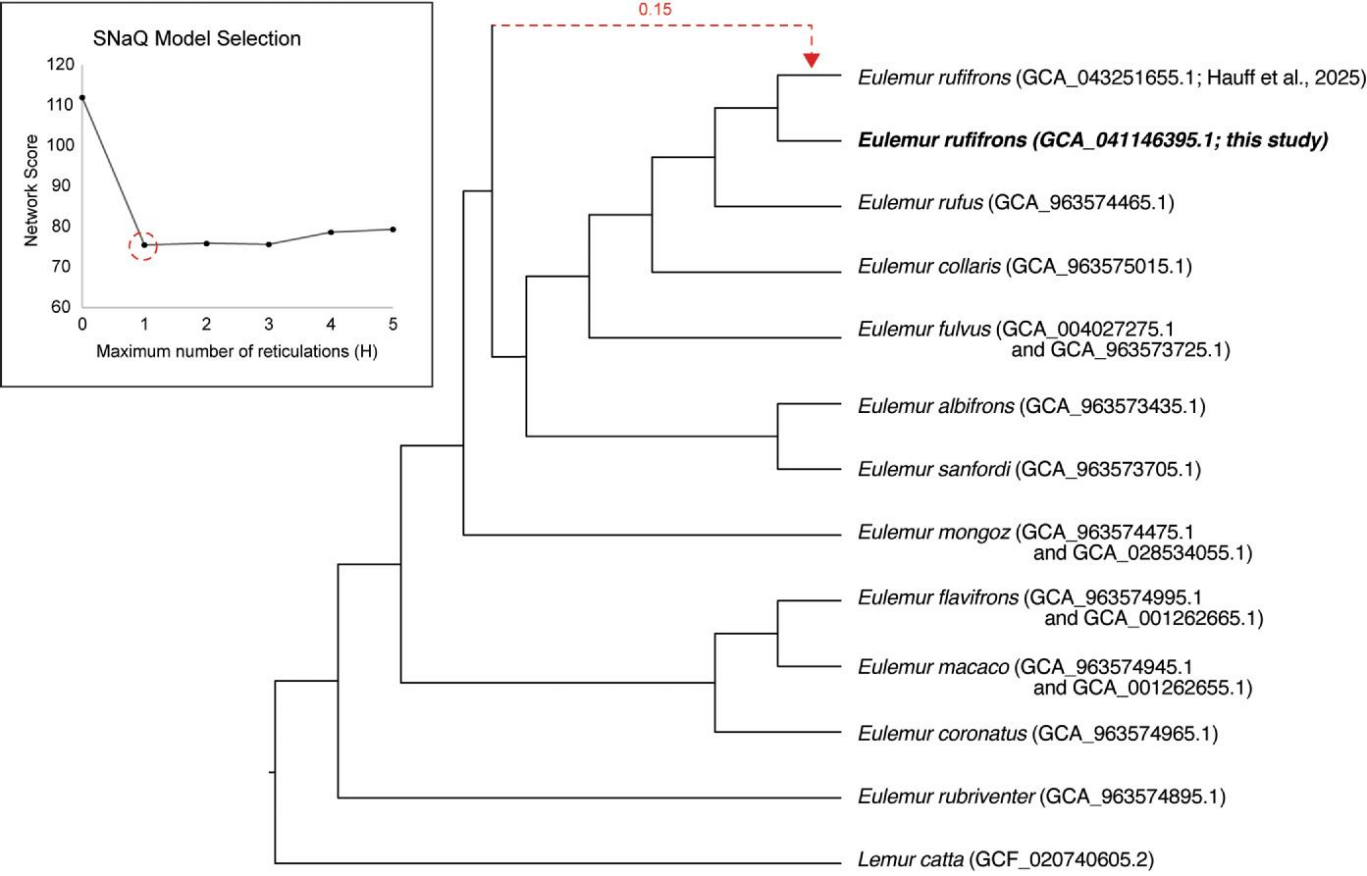
A phylogenetic network estimated using SNaQ. The inset shows network scores of each evaluated model (*H* = 0 to 5). The top-scoring model (*H* = 1) is circled, and the phylogenetic network produced by this model is shown on the right. The dashed line in the network identifies a reticulation event involving an ancestral brown lemur lineage and the *E. rufifrons* genome assembly generated by Hauff et al. (2025).

The top model chosen by SNaQ had 1 hybrid edge (*H* = 1), although we note that models with 2, 3, 4, or 5 hybrid edges were also well supported (Fig. 5). The network estimated using the *H* = 1 model shows a hybrid edge between the previously published *E. rufifrons* genome (GCA_043251655) and an unknown, unsampled taxon sister to the *E. fulvus* species complex. In this network, the 2 *E. rufifrons* individuals form a monophyletic group. All other relationships were identical between our SNaQ and ASTRAL trees.

## Discussion

Here, we presented a chromosome-scale assembly of *E. rufifrons* generated from the combination of long-read DNA sequence data (PacBio) and chromatin conformation capture data (Hi-C). It is the most complete genome to date for the family Lemuridae, edging out the existing ring-tailed lemur (*L. catta*) genome based on BUSCO metrics (Fig. 2). Our assembly results are similar to those of *E. mongoz*, the other chromosome-level assembly for the genus (also assembled using Hi-C data), and match chromosome number expectations based on karyotypic data (Hamilton and Buettner-Janusch 1977; Hamilton et al. 1980). Notably, our chromosomes were labeled according to names assigned to the *E. mongoz* genome, which did not label sex chromosomes. Thus, it is likely that our genome assembly includes sequence data from the X chromosome, but this could not be accurately identified. Nonetheless, an autosomal synteny comparison of our *E. rufifrons* genome with the *E. mongoz* genome revealed a remarkable level of structural conservation in terms of the locations of single-copy genes along chromosomes (Fig. 3), despite ∼4 million years of divergence between these species (Everson et al. 2025).

One intriguing result of our phylogenetic analyses was the inconsistent placement of our genome as either sister to *E. rufus* (Fig. 4) or as sister to another recently published *E. rufifrons* genome (Fig. 5). This uncertainty may not be surprising given that *E. rufifrons* and *E. rufus* were previously considered a single species (Mittermeier et al. 2008) and population genetic data have identified shared variation between the 2 species (Markolf et al. 2013). We again note that pedigree information for Redbay was incomplete and morphological information was not overtly consistent with *E. rufifrons* or *E. rufus*, although we believe that her pelage color was consistent with western populations of *E. rufifrons*. It is also worth noting that the Hauff et al. (2025)  *E. rufifrons* genome, which was sampled from an eastern population in Ranomafana National Park, is smaller in size (2.2 Gb) and chromosome number (n = 28) compared to our genome. Together, these results suggest that a comprehensive reassessment of species boundaries and *Eulemur* taxonomy using population-level data may be warranted.

Overall, evolutionary analyses of our genome with other *Eulemur* genomic data were consistent with the idea that *Eulemur* is a young radiation with incomplete species boundaries (Markolf and Kappeler 2013; Everson et al. 2023, Everson et al. 2025), particularly among members of the brown lemur complex. The conserved genome structure we identified between *E. rufifrons* and *E. mongoz* may provide at least part of the explanation for rampant hybridization across the genus, as it suggests that chromosomal architecture likely does not serve as a prezygotic barrier to reproduction. Our growing understanding of the history of introgressive hybridization across *Eulemur* sets the stage for expanded use of genome-scale data in the study of the clade's diversification. This could include the opportunity to explore how evolutionary processes have operated across the genome during and after speciation and diversification. For example, are there differential patterns of selection or introgression in gene regions between species? Or are there signatures of adaptive introgression? By expanding the set of complete and robust chromosomal assemblies available for representatives of the *Eulemur* clade, we have provided a jumping-off point for using whole-genome resequencing population data sets to effectively answer these questions.

## Supplementary Material

jkaf213_Supplementary_Data

## Data Availability

Unprocessed sequence data have been archived to NCBI Sequence Read Archive under Bioproject PRJNA1104249. The assembled Refseq genome can be found under accession GCF_041146395.1 along with NCBI Annotation Release 100. All bash scripts and R code used in this study can be found at https://github.com/Andrew-N-Black/Eulemur_genome. Supplemental material available at G3 online.
