# Supplementary material for: An annotated chromosome-level genome for the red-fronted brown lemur (Eulemur rufifrons) sheds light on brown lemur evolution
Source: G3 (Bethesda). 2025 Sep 11;15(11):jkaf213. doi: 10.1093/g3journal/jkaf213 (PMC12609170; doi:10.1093/g3journal/jkaf213)
Supplement: jkaf213_Supplementary_Data [file jkaf213_supplementary_data.zip › Supplemental_Figure_Legends_G3-2025-405969.docx]

**Supplemental Figure Legends**

Figure S1. Contact matrix illustrating the Hi-C optical map for the Redbay *Eulemur rufifrons* assembly (GCA_041146395.1).

Figure S2. Chromosomal (n=30) D-Genies (Cabanettes and Klopp, 2018) dotplot between the Redbay *Eulemur rufifrons* (GCA_041146395.1) assembly vs the *E. mongoz* (GCA_028534055.1) assembly.

Figure S3. Scatterplot illustrating the correlation in chromosome length (nt) between the Redbay *Eulemur rufifrons* (GCA_041146395.1) assembly and the (Target) *E. mongoz* (GCA_028534055.1) assembly. The diagonal blue line depicts a 1:1 ratio between chromosome length for the length of the n=30 chromosome.

Figure S4. Counts of telomeric repeats in 10-kb windows across each identified chromosome, as estimated by the software tidk using the canonical mammalian repeat (TTAGGG)_n_. The y-axis scale is scaled individually for each chromosome. Stars indicate locations where a high concentration of telomeric repeats have been identified on that end of the chromosome.
