# Supplementary figures and images for: An annotated chromosome-level genome for the red-fronted brown lemur (Eulemur rufifrons) sheds light on brown lemur evolution
Source: G3 (Bethesda). 2025 Sep 11;15(11):jkaf213. doi: 10.1093/g3journal/jkaf213 (PMC12609170; doi:10.1093/g3journal/jkaf213)

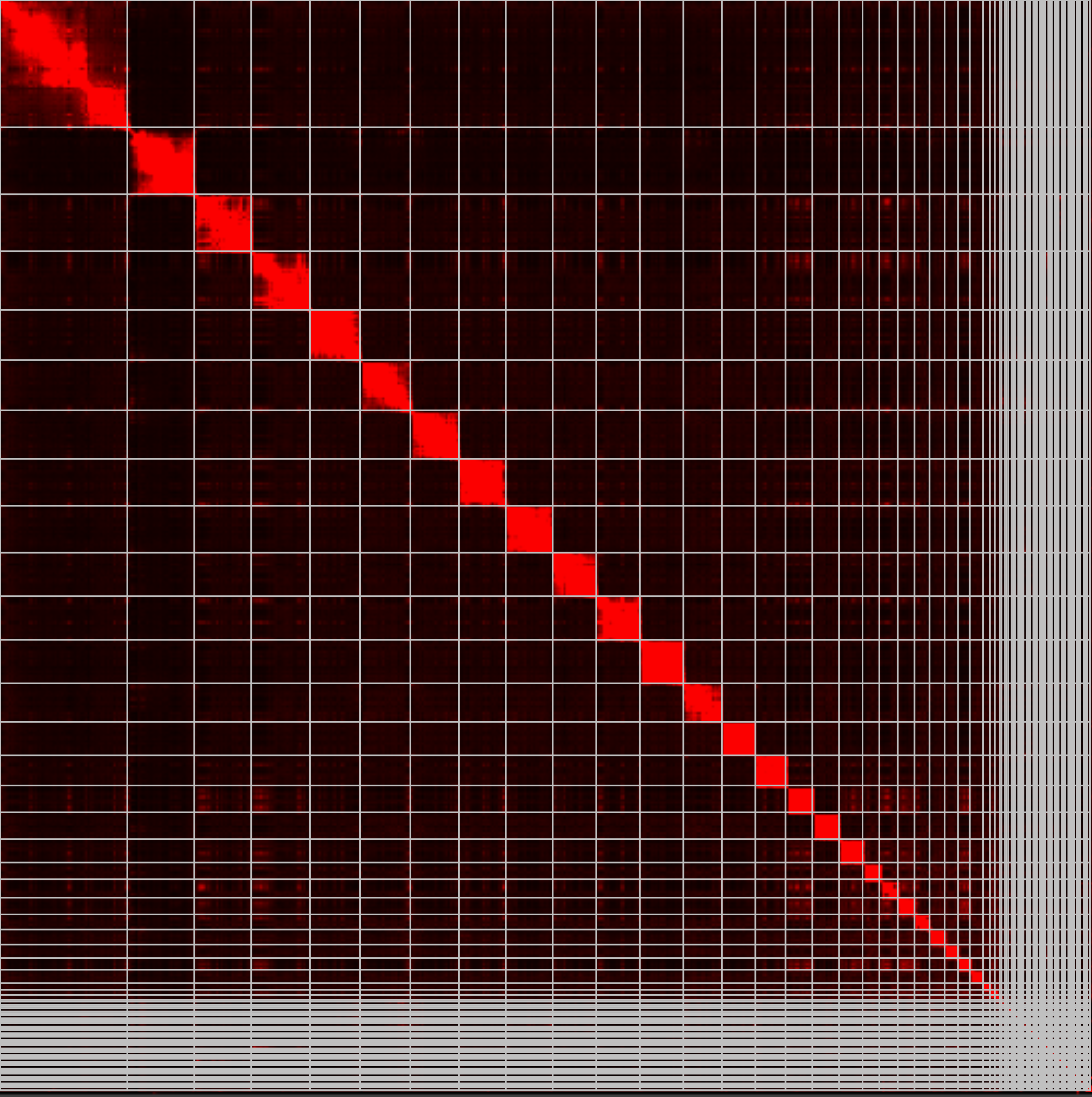

Supplement: jkaf213_Supplementary_Data [file jkaf213_supplementary_data.zip › Figure_S1_G3-2025-405969.png]

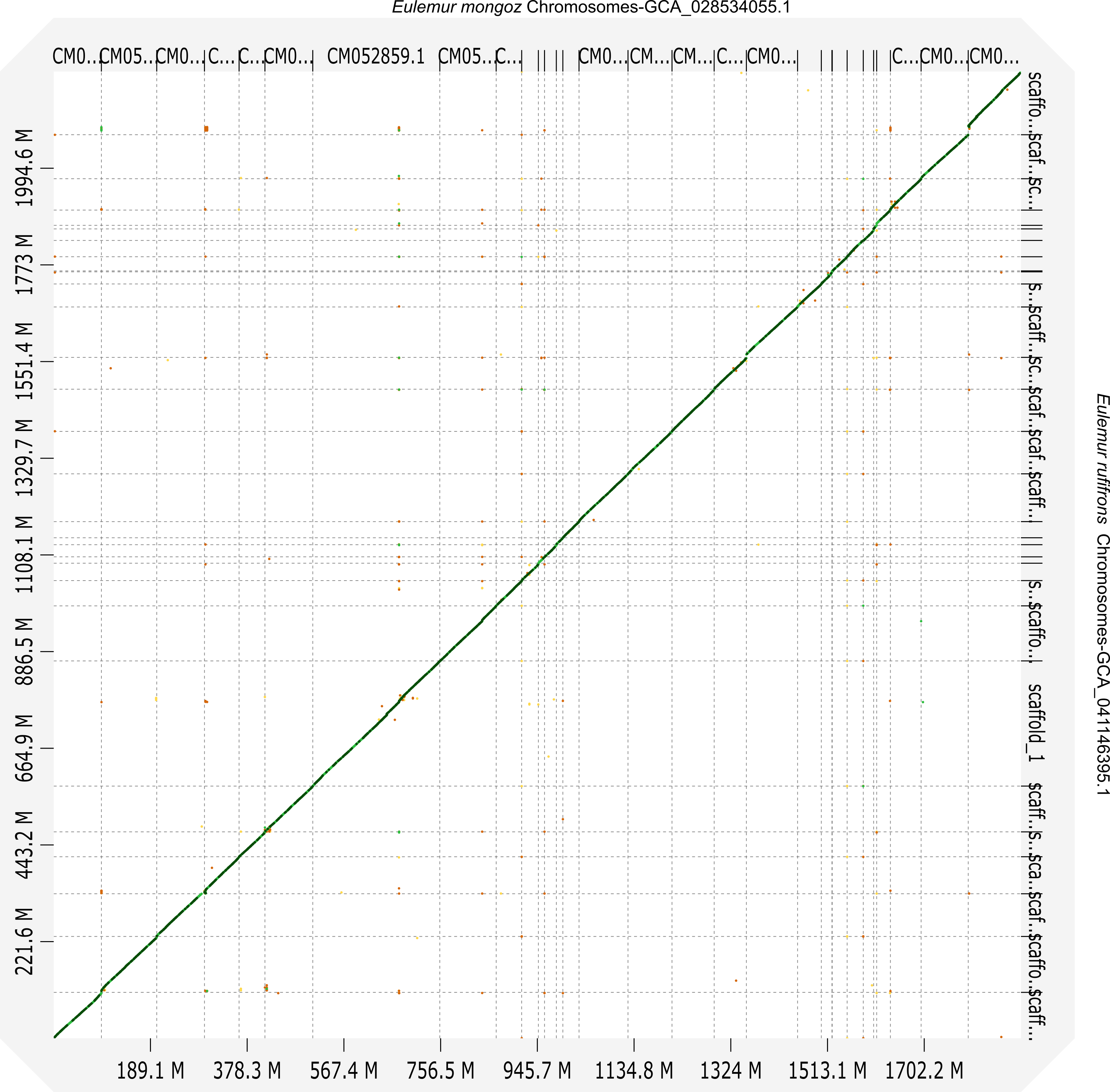

Supplement: jkaf213_Supplementary_Data [file jkaf213_supplementary_data.zip › Figure_S2_G3-2025-405969.png]

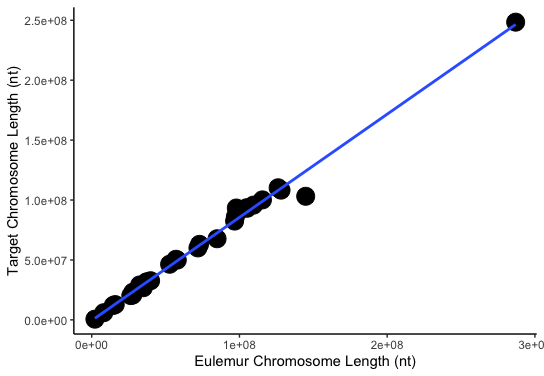

Supplement: jkaf213_Supplementary_Data [file jkaf213_supplementary_data.zip › Figure_S3_G3-2025-405969.png]

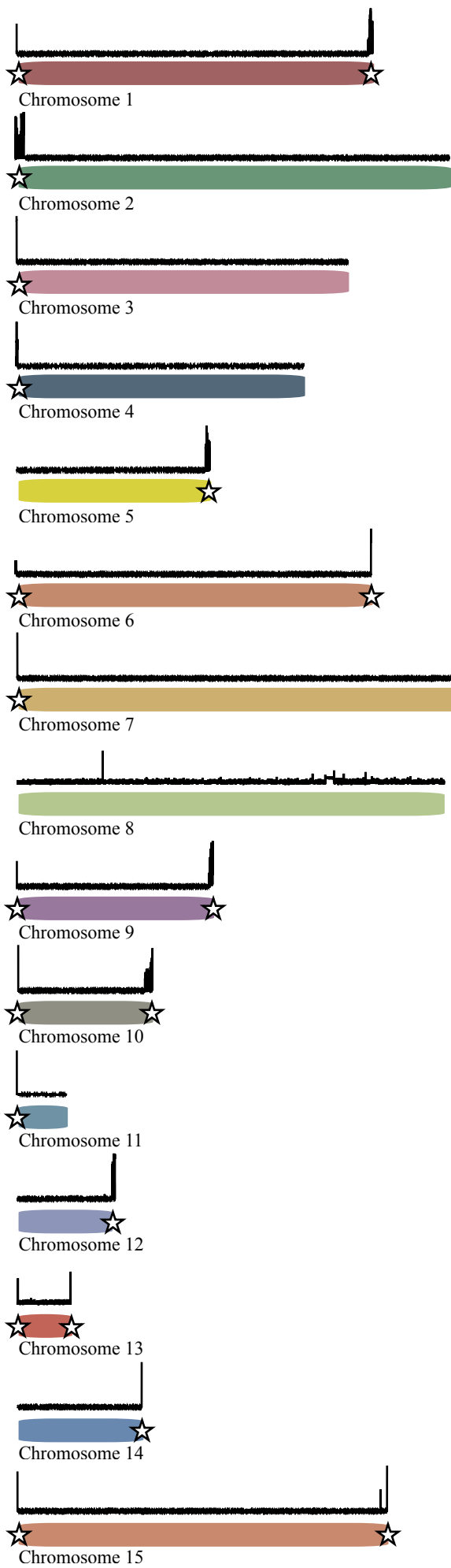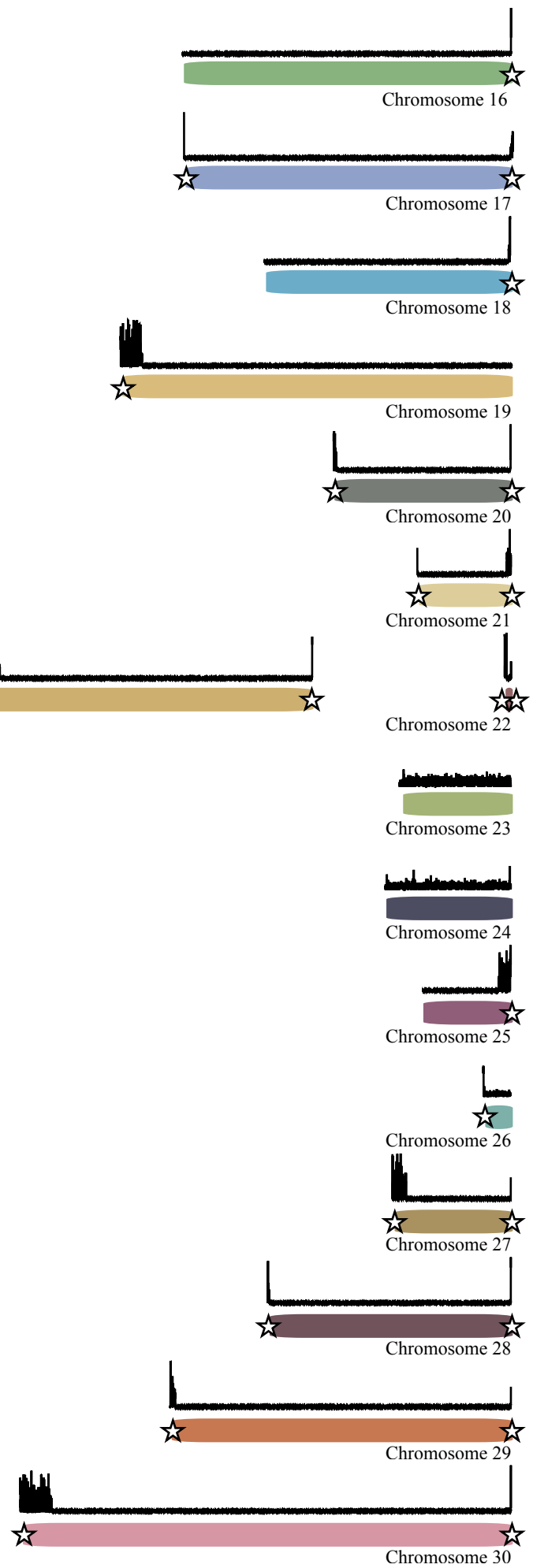

Supplement: jkaf213_Supplementary_Data [file jkaf213_supplementary_data.zip › Figure_S4_G3-2025-405969.pdf]
